# Supplementary material for: Molecular characterization of the N-terminal half of TasA during amyloid-like assembly and its contribution to Bacillus subtilis biofilm formation
Source: NPJ Biofilms Microbiomes. 2023 Sep 22;9:68. doi: 10.1038/s41522-023-00437-w (PMC10516879; doi:10.1038/s41522-023-00437-w)
Supplement: Supplementary file 1 — Supplementary_files_merged [file 41522_2023_437_MOESM1_ESM.pdf]

Molecular characterization of the N-terminal half of TasA during amyloid-like assembly and its contribution to *Bacillus subtilis* biofilm formation

Jesús Cámara-Almirón, Laura Domínguez-García, Nadia El Mammeri, Alons Lends, Birgit Habenstein, Antonio de Vicente, Antoine Loquet, Diego Romero

Supplementary files

-Supplementary figures 1-4

-Supplementary tables 1 and 2

-Source data for gels and blots

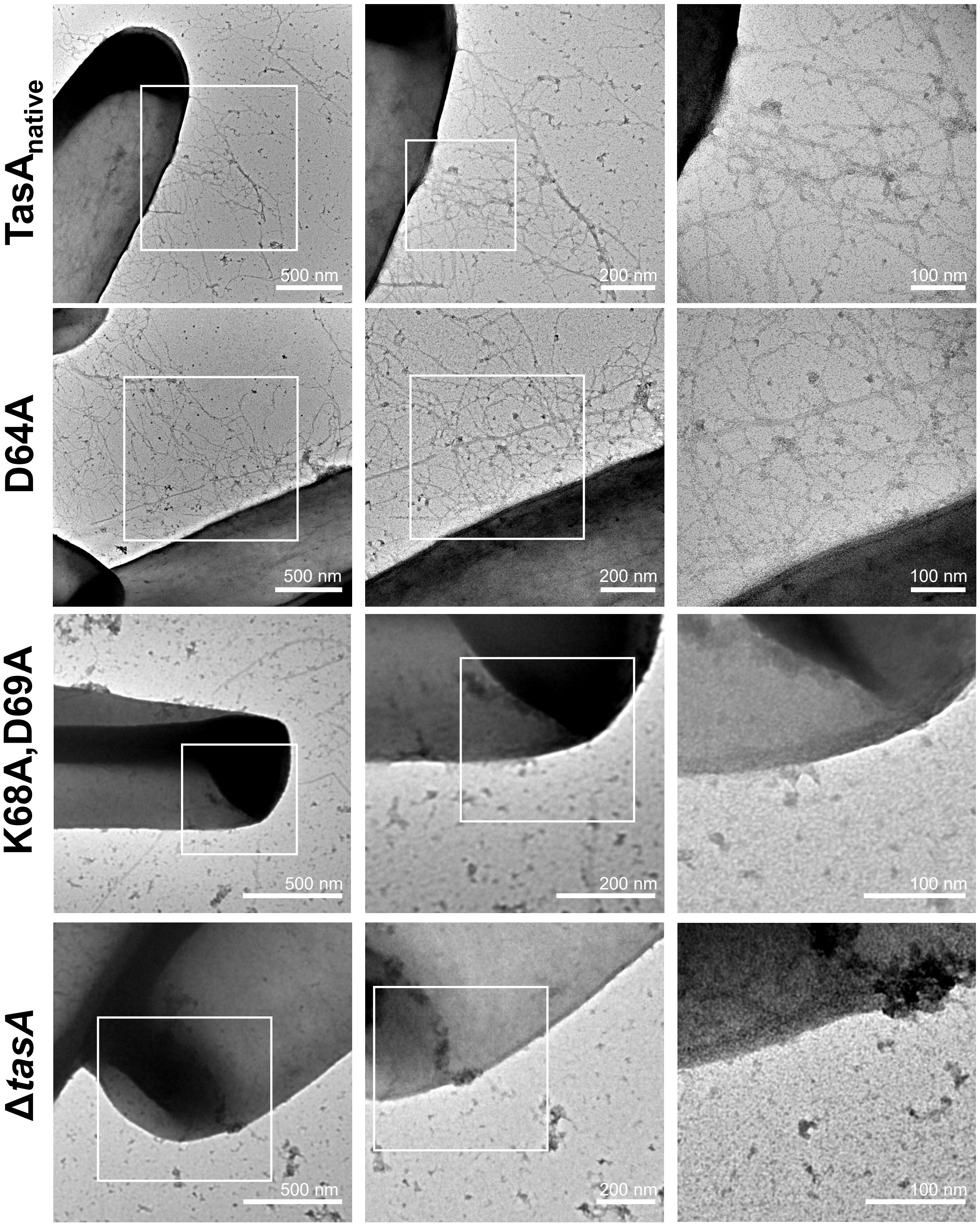

Supplementary figure 1. Transmission Electron Microscopy Images of Cells Carrying the Native Protein or the Protein Variants. Transmission electron microscopy images of negatively stained samples of cells carrying the native protein, the D64A or K68AD69A alleles or Δ*tasA* growing under biofilm inducing conditions in MSgg medium. White squares indicate areas of the images that have been zoomed in. Scale bars = 500 nm (left), 200 nm (middle) and 100 nm (right).

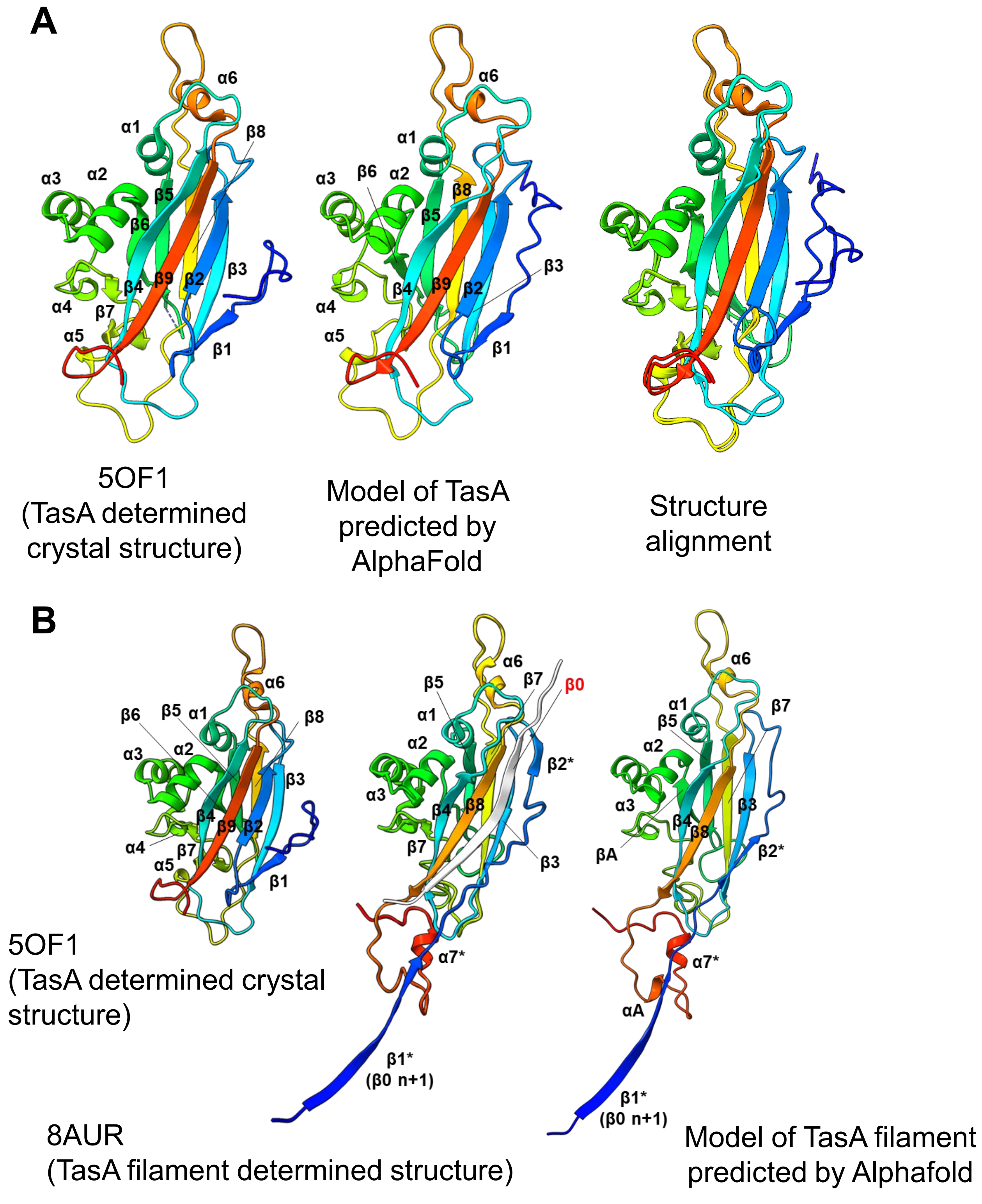

Supplementary figure 2. Structure comparison between publicly available TasA structures and predicted models. A) Structure comparison between the crystal structure of TasA (5OF1) and the model predicted by AlphaFold. The numbering in the scheme follow the representation described for the crystal structure of TasA in its monomeric form published by Diehl, A. et al. (2018). The image on the right shows the superimposition of the two structures. The coloring scheme indicates the position of the residue in the sequence of the protein, where blue indicates the N-terminal and red indicates the C-terminal. Warmer colors indicate proximity to the C-terminal end. **B)** Structure comparison between the crystal structure of TasA (5OF1) and the subunits of the TasA filament determined structure (8AUR) (middle) or AlphaFold prediction (right). The coloring scheme indicates the position of the residue in the sequence of the protein, where blue indicates the N-terminal and red indicates the C-terminal. Warmer colors indicate proximity to the C-terminal end. The numbering in the scheme of the subunit models references the numbers used for the monomer structure, same secondary structure elements share the same numbering in all the models present in the figure.

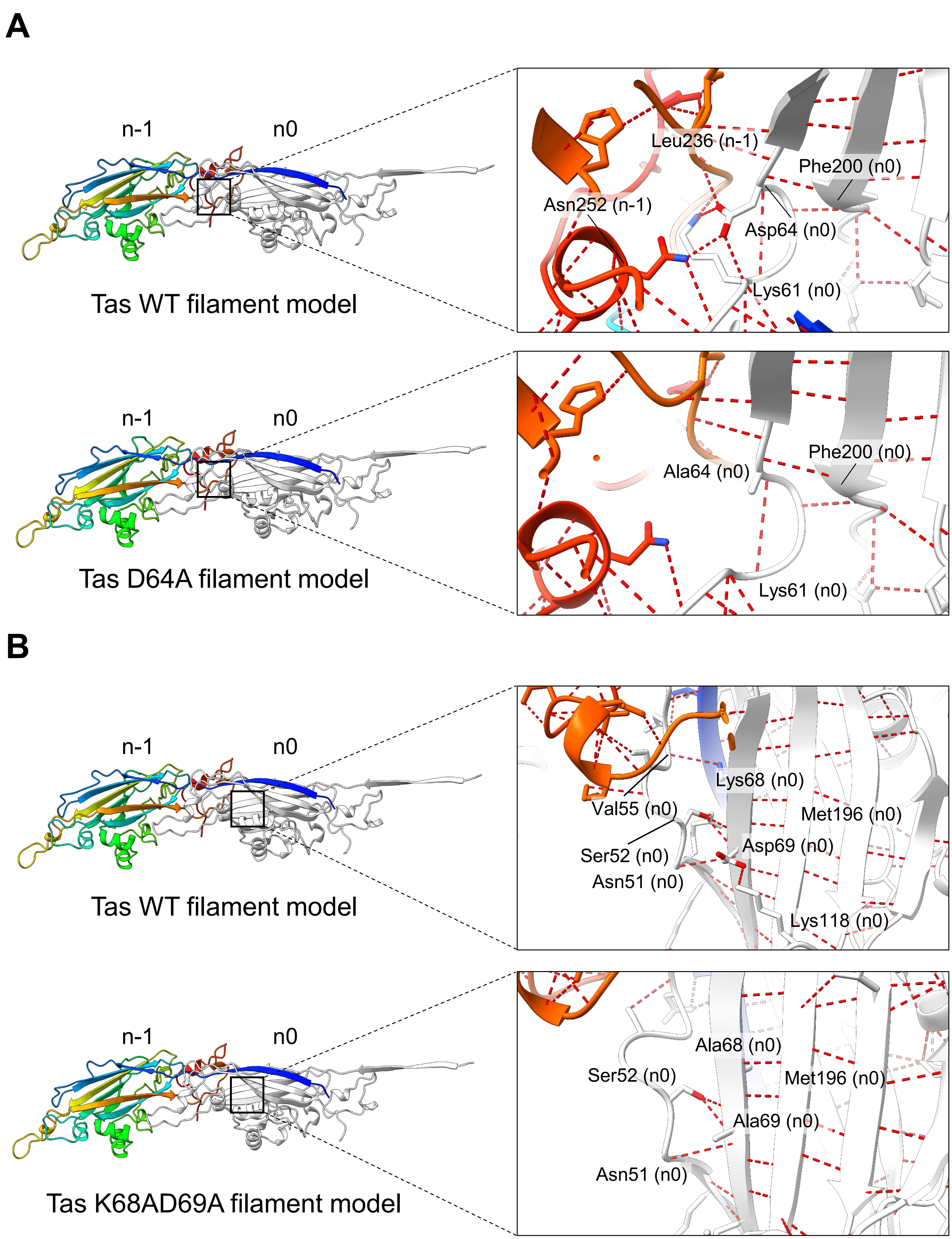

Supplementary figure 3. Structure comparison between the filament models of WT TasA and TasA D64A (A) or WT TasA and TasA K68AD69A (B). The coloring scheme in the n-1 subunit indicates the position of the residue in the sequence of the protein, where blue indicates the N-terminal and red indicates the C-terminal. Warmer colors indicate proximity to the C-terminal end. White color indicates the n0 subunit in both cases.

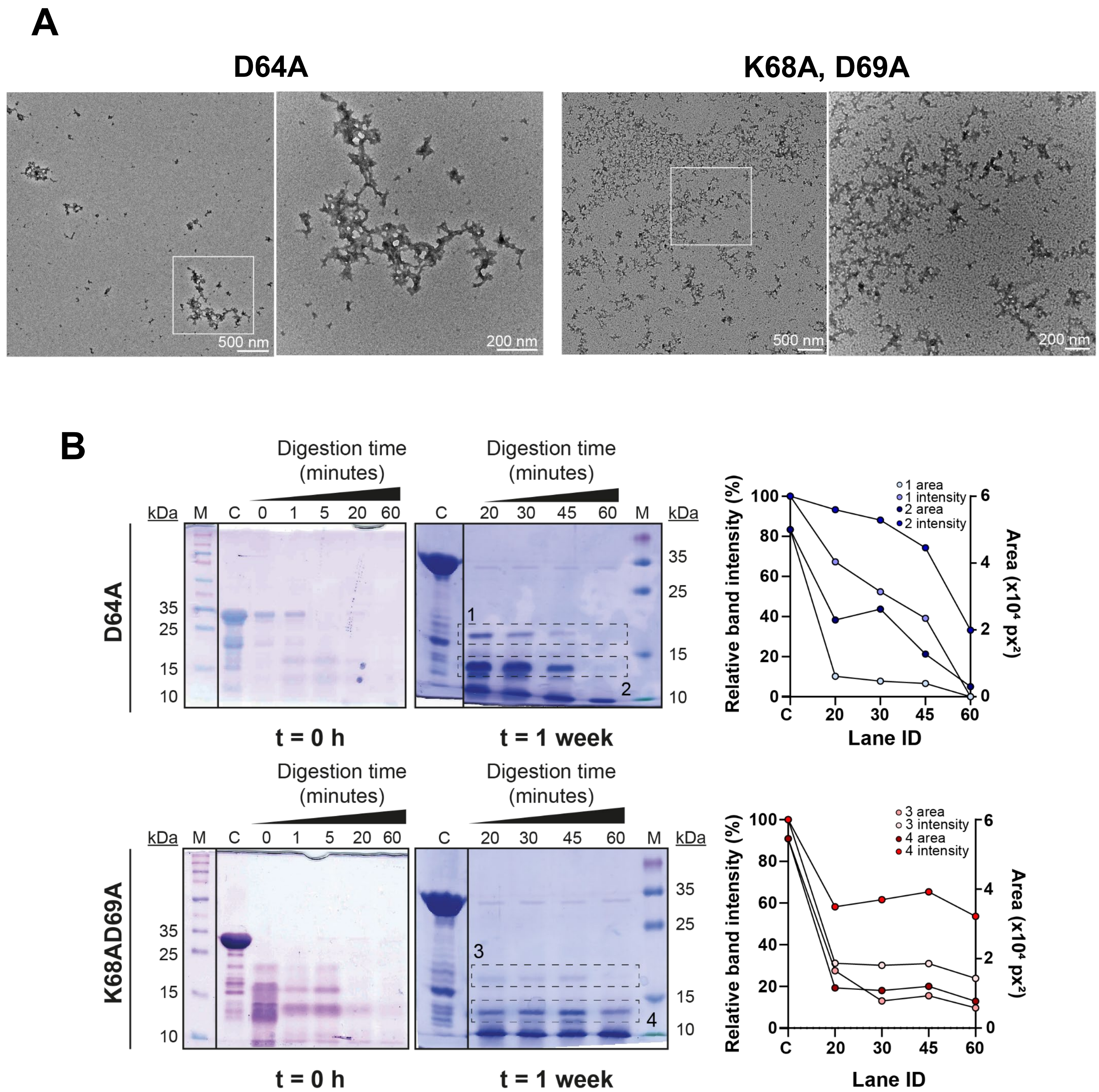

Supplementary figure 4. Characterization of TasA D64A and K68AD69A samples at 0 h or 1 week after buffer exchange and incubation at 30 °C. A) Transmission electron micrographs of negatively stained TasA D64A (left) or K68AD69A samples (right) of 0 h of incubation after buffer exchange. White squares over the images indicate the areas that were zoomed in during the imaging. The scale bars are, from left to right, 500 nm and 200 nm. B) Top: Coomassie stained SDS-PAGE gel of TasA D64A assembled for 0 h (left) or 1 week (middle) after buffer exchange and digested with proteinase K at different time points. M lane indicates the molecular marker. C indicates the untreated control. Each lane contains a sample corresponding to a specific digestion time. SDS-PAGE gel images have been cropped and spliced for illustrative purposes. The lines over the gel images indicates the boundaries of the image splicing. The two slices in the two different gel images are derived from a single gel, respectively. Dashed rectangles over the gel images indicate protease resistant bands that have been quantified in the right graph. Right. Quantification of the relative intensity and area of the bands marked in the gel. Bottom. Coomassie stained SDS-PAGE gel of TasA K68AD69A assembled for 0 h (left) or 1 week (middle) after buffer exchange and digested with proteinase K at different time points. M lane indicates the molecular marker. C indicates the untreated control. Each lane contains a sample corresponding to a specific digestion time. SDS-PAGE gel images have been cropped and spliced for illustrative purposes. The lines over the gel images indicates the boundaries of the image splicing. The two slices in the two different gel images are derived from a single gel. Dashed rectangles over the gel images indicate protease resistant bands that have been quantified in the right graph. Right. Quantification of the relative intensity and area of the bands marked in the gel.

Supplementary table 1. Bacterial strains used in this study

Bacterial strain	Genotype	Source
Bacillus subtilis 168	Laboratory strain	Laboratory collection
Bacillus subtilis NCIB3610	Wild type. Undomesticated strain	Laboratory collection
CA017	*Bacillus subtilis* NCIB3610 *tasA::km*	(Romero *et al*., 2010) (15)
SSB488	*Bacillus subtilis* NCIB3610<br>*epsA-O::tet*	(Branda *et al*., 2006) (13)
SSB149	*Bacillus subtilis* NCIB3610<br>*(tapA-sipW-tasA)::spc*	(Branda *et al*., 2006) (13)
JC70	*Bacillus subtilis* NCIB3610<br>*(tapA-sipW-tasA)::spc*<br>*lacA::(tapA-sipW-*$tasA_{native}$*) (mls)*	(Cámara-Almirón *et al*., 2020) (25)
JC81	*Bacillus subtilis* NCIB3610<br>*(tapA-sipW-tasA)::spc*<br>*lacA::(tapA-sipW-*$tasA_{(Lys68Ala,\ Asp69Ala)}$*) (mls)*	(Cámara-Almirón *et al*., 2020) (25)
JC78	*Bacillus subtilis* NCIB3610<br>*(tapA-sipW-tasA)::spc*<br>*lacA::(tapA-sipW-*$tasA_{Asp64Ala}$*) (mls)*	This work
JC72	*Bacillus subtilis* NCIB3610<br>*(tapA-sipW-tasA)::spc*<br>*lacA::(tapA-sipW-*$tasA_{\Delta 82\text{-}88}$*) (mls)*	This work
JC75	*Bacillus subtilis* NCIB3610<br>*(tapA-sipW-tasA)::spc*<br>*lacA::(tapA-sipW-*$tasA_{\Delta 108\text{-}116}$*) (mls)*	This work
JC76	*Bacillus subtilis* NCIB3610<br>*(tapA-sipW-tasA)::spc*<br>*lacA::(tapA-sipW-*$tasA_{Glu82Ala}$*) (mls)*	This work
JC77	*Bacillus subtilis* NCIB3610<br>*(tapA-sipW-tasA)::spc*<br>*lacA::(tapA-sipW-*$tasA_{Phe72Ala}$*) (mls)*	This work
JC80	*Bacillus subtilis* NCIB3610<br>*(tapA-sipW-tasA)::spc*<br>*lacA::(tapA-sipW-*$tasA_{Lys35Ala,\ Asp36Ala}$*) (mls)*	This work
JC82	*Bacillus subtilis* NCIB3610<br>*(tapA-sipW-tasA)::spc*<br>*lacA::(tapA-sipW-*$tasA_{Gly96Ala}$*) (mls)*	This work
JC226	*Bacillus subtilis* NCIB3610<br>*(tapA-sipW-tasA)::spc*<br>*lacA::(tapA-sipW-*$tasA_{native}$*) (mls)*<br>*epsA-O::tet*	This work
JC228	*Bacillus subtilis* NCIB3610<br>*(tapA-sipW-tasA)::spc*<br>*lacA::(tapA-sipW-*$tasA_{Asp64Ala}$*) (mls)*<br>*epsA-O::tet*	This work
JC231	*Bacillus subtilis* NCIB3610<br>*(tapA-sipW-tasA)::spc*<br>*lacA::(tapA-sipW-*$tasA_{(Lys68Ala,\ Asp69Ala)}$*) (mls)*<br>*epsA-O::tet*	This work
JC118	*E. coli* DH5α<br>*pET22b-*$TasA_{K35 - K144}$ *(RcTasA)*	This work
JC104	*E. coli* DH5α<br>*pET22b-*$TasA_{D64A}$	This work

JC106	*E. coli* DH5α<br>*pET22b-TasA*$_{K68A,\ D69A}$	This work

Supplementary table 2. Primers used in this study

Name	Sequence (5' – 3')	Purpose
Am_core_exp_C_Rv	aaaaactcgagtttagcagacatcaaatacaagtc	Cloning of the rigid core of TasA into pET22b
Am_core_exp_C_Fw	aaaaacatatgaaggatgctacttttgcatcagg	Cloning of the rigid core of TasA into pET22b
TasA_Exp_C_NdeI_F	aaaaacatatggcatttaacgacattaaatcaaa	Cloning of TasA D64A and TasA K68A, D69A into pET22b
TasA_Exp_C_XhoI_R	aaaaactcgagatttttatcctcgctatgcgcttt	Cloning of TasA D64A and TasA K68A, D69A into pET22b
del82-88	caatttgaaaataacggatcacttgcgatcaaatatggagattttaaagcaaac	Deletion of amino acids 82-86 from the TasA sequence by site-directed mutagenesis
del82-88-antisense	gtttgctttaaaatctccatatttgatcgcaagtgatccgttattttcaaattg	Deletion of amino acids 82-86 from the TasA sequence by site-directed mutagenesis
del108-116	cagaagatttcctcagcggaaaagagggcggcaatg	Deletion of amino acids 108-116 from the TasA sequence by site-directed mutagenesis
del108-116-antisense	cattgccgccctcttttccgctgaggaaatcttctg	Deletion of amino acids 108-116 from the TasA sequence by site-directed mutagenesis
KD_AA_35-36	ctgatgcaaaagtagcagccgctgatttaatgtcgttaaatgctgcccatgttcc	Amino acid substitution of amino acids 35 and 36 of TasA sequence by alanines using site-directed mutagenesis
KD_AA_35-36_antisense	ggaacatgggcagcatttaacgacattaaatcagcggctgctacttttgcatcag	Amino acid substitution of amino acids 35 and 36 of TasA sequence by alanines using site-directed mutagenesis
F_A_72	gtgatccgttattttcagcttggaaatcctttgtcaacttatctcccggc	Amino acid substitution of amino acid 72 of TasA sequence by alanine using site-directed mutagenesis
F_A_72_antisense	gccgggagataagttgacaaaggatttccaagctgaaaataacggatcac	Amino acid substitution of amino acid 72 of TasA sequence by alanine

		using site-directed mutagenesis
E_A_82	attaagcgccattagaactgctttgatcgcaagtgatcc	Amino acid substitution of amino acid 82 of TasA sequence by alanine using site-directed mutagenesis
E_A_82_antisense	ggatcacttgcgatcaaagcagttctaatggcgcttaat	Amino acid substitution of amino acid 82 of TasA sequence by alanine using site-directed mutagenesis
G_A_96	ggagatgtattgctgccggcgtttgctttaaaatctc	Amino acid substitution of amino acid 96 of TasA sequence by alanine using site-directed mutagenesis
G_A_96_antisense	gagattttaaagcaaacgccggcagcaatacatctcc	Amino acid substitution of amino acid 96 of TasA sequence by alanine using site-directed mutagenesis
D_A_64	ggaaatcctttgtcaacttagctcccggctttagatttgat	Amino acid substitution of amino acid 64 of TasA sequence by alanine using site-directed mutagenesis
D_A_64_antisense	atcaaatctaaagccgggagctaagttgacaaaggatttcc	Amino acid substitution of amino acid 64 of TasA sequence by alanine using site-directed mutagenesis

Source data for gels and blots: uncropped gels and blots images

Figure 2

Figure 2E

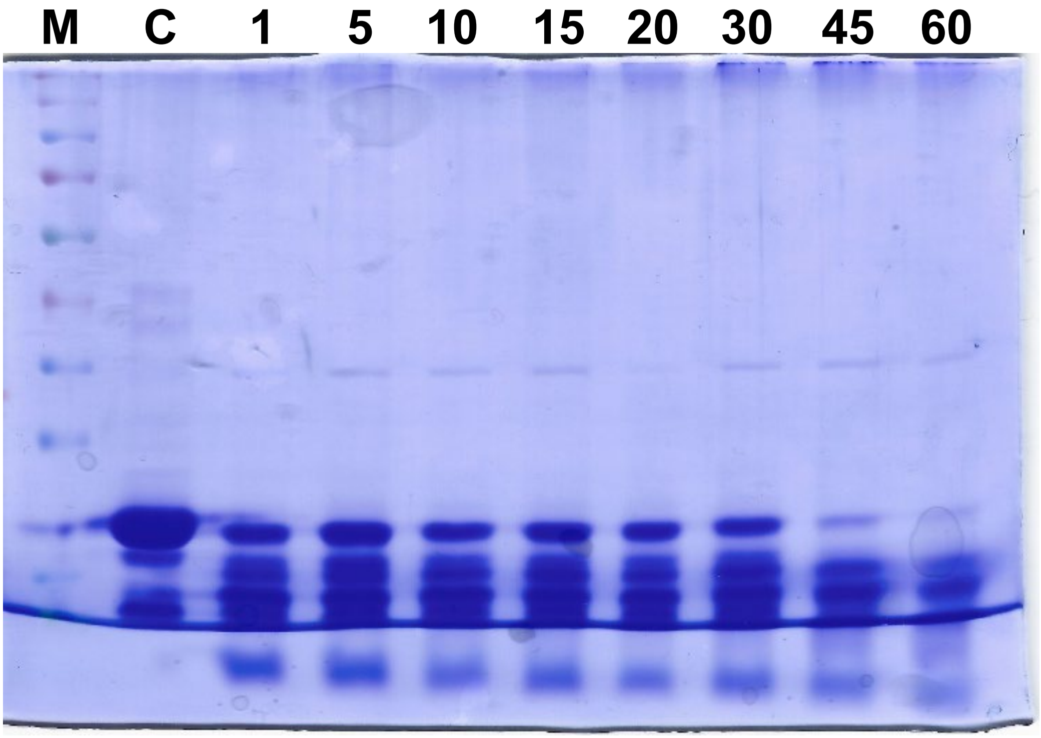

Lanes were loaded with samples corresponding to one digestion time

Figure 2F

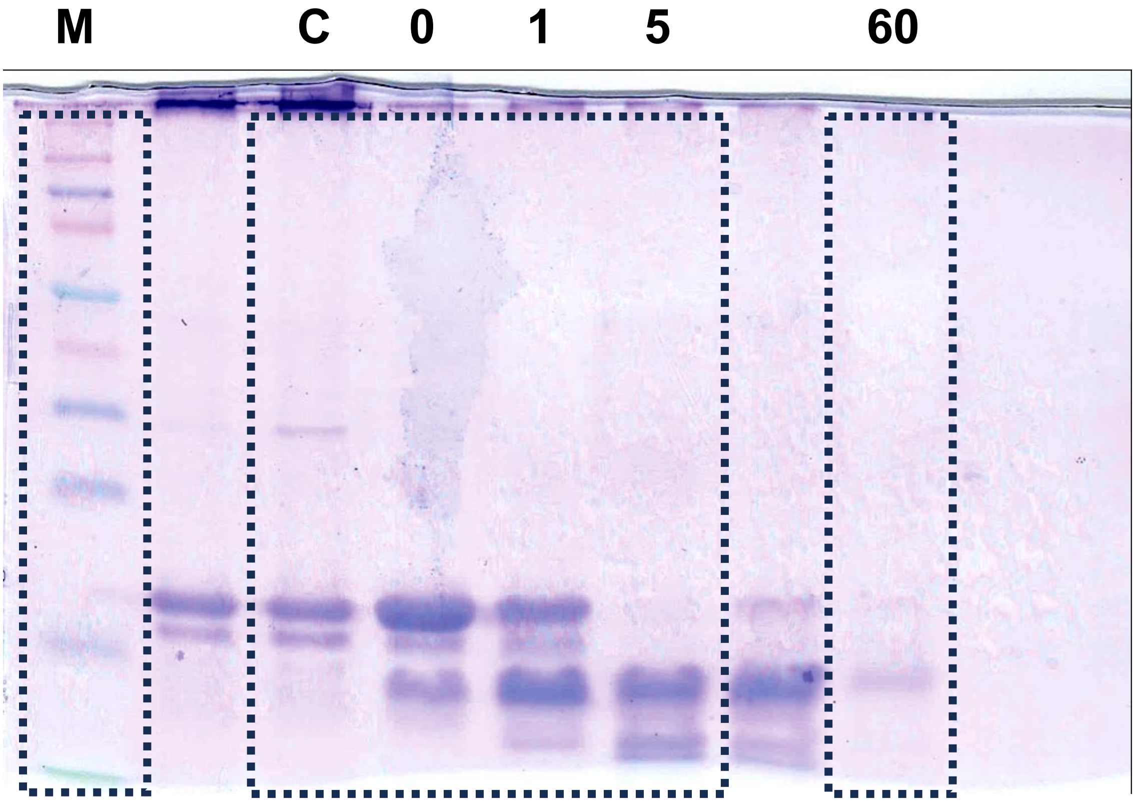

Lanes were loaded with samples correspondi ng to one digestion time

Figure 3C top

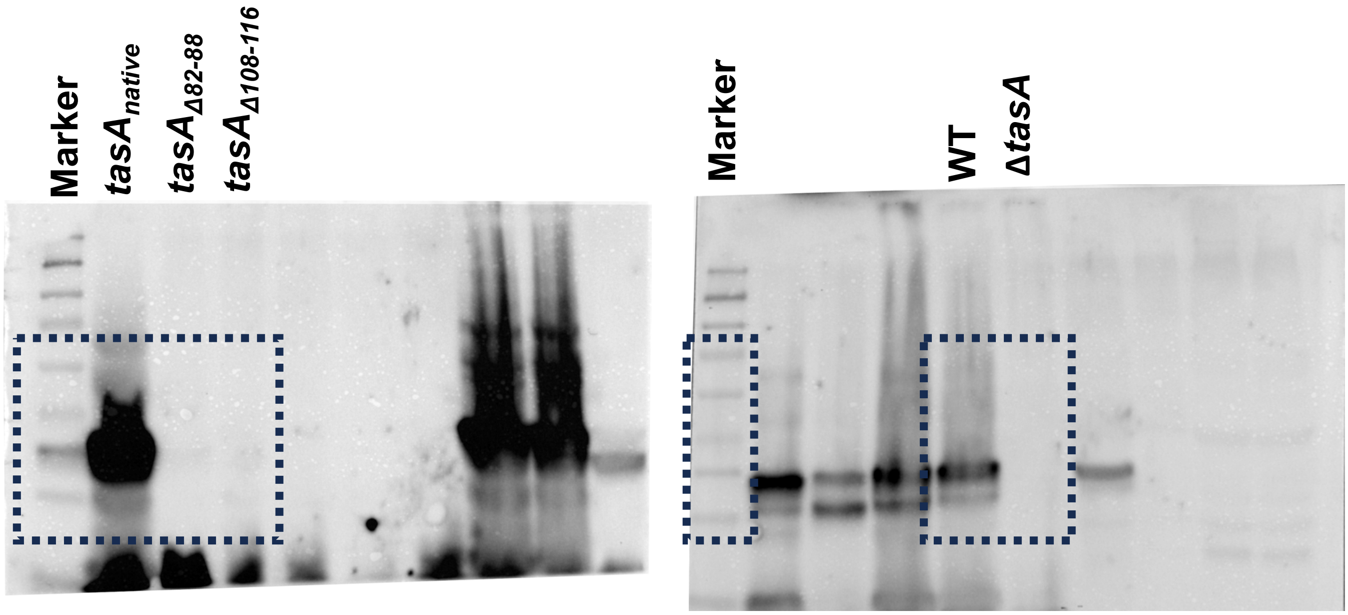

Figure 3C middle

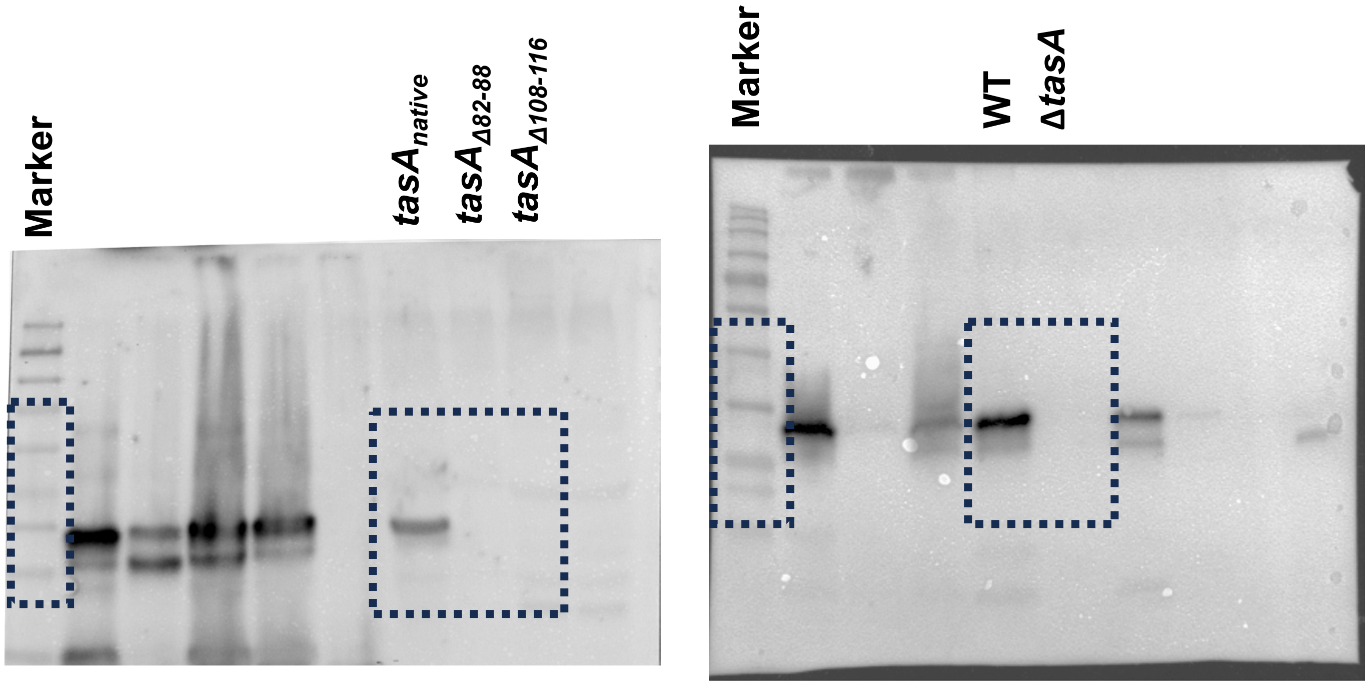

Figure 3C bottom

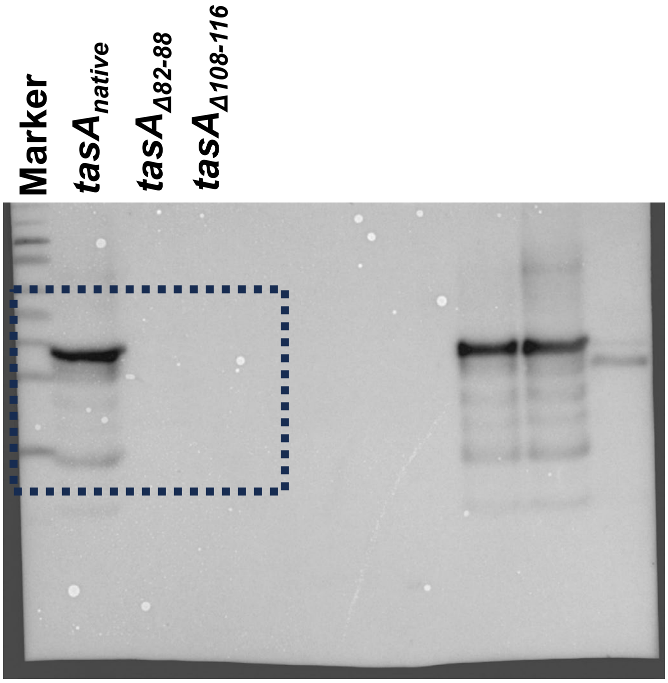

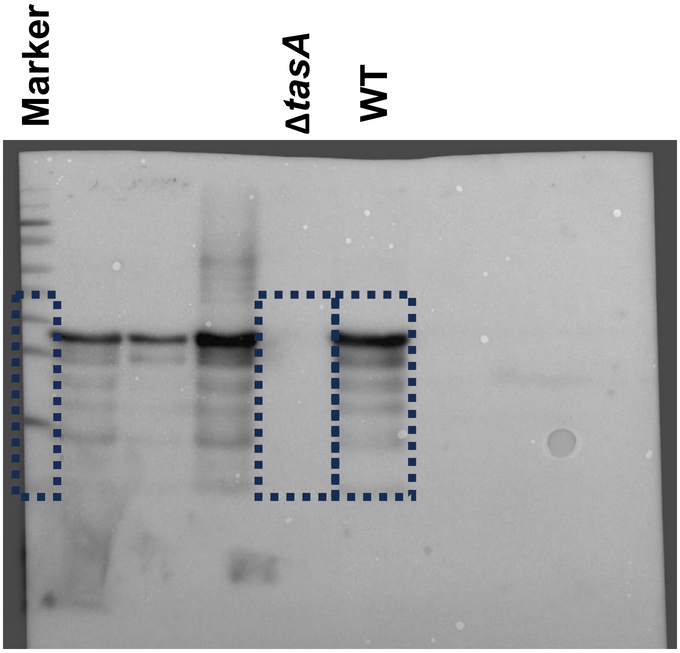

Figure 4B top

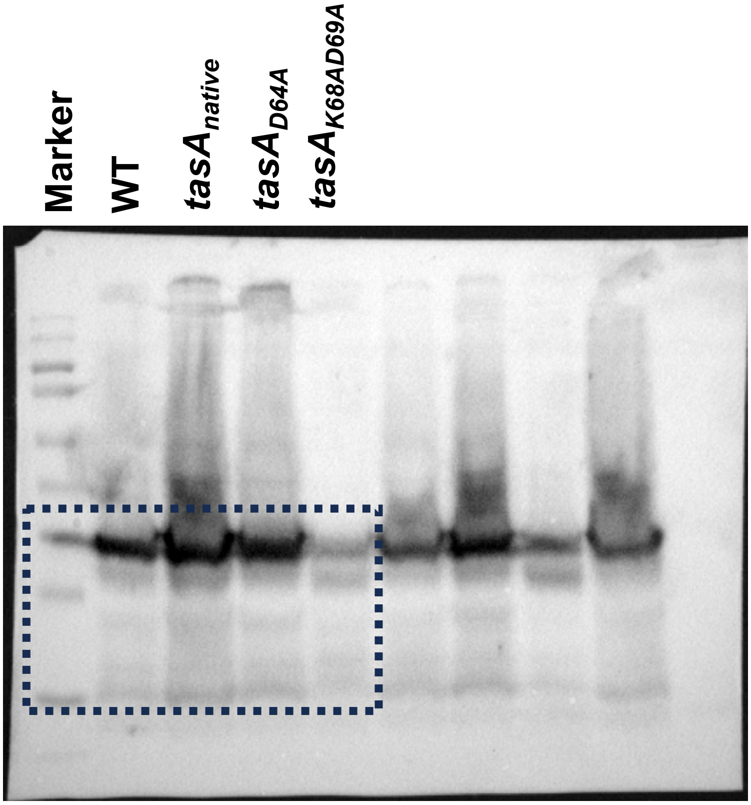

Figure 4B middle

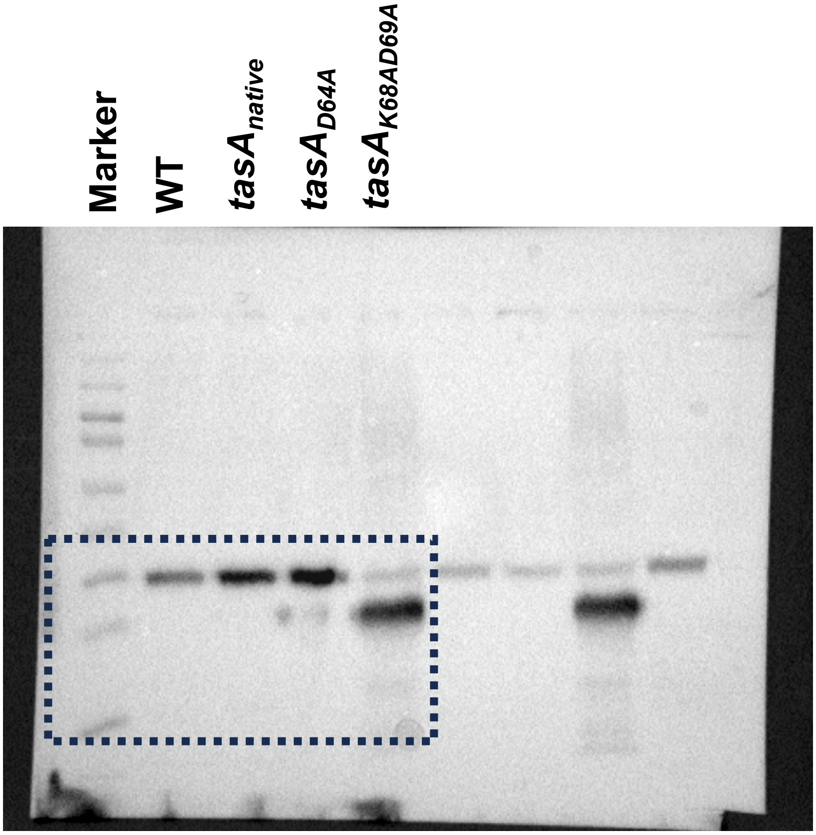

Figure 4C bottom

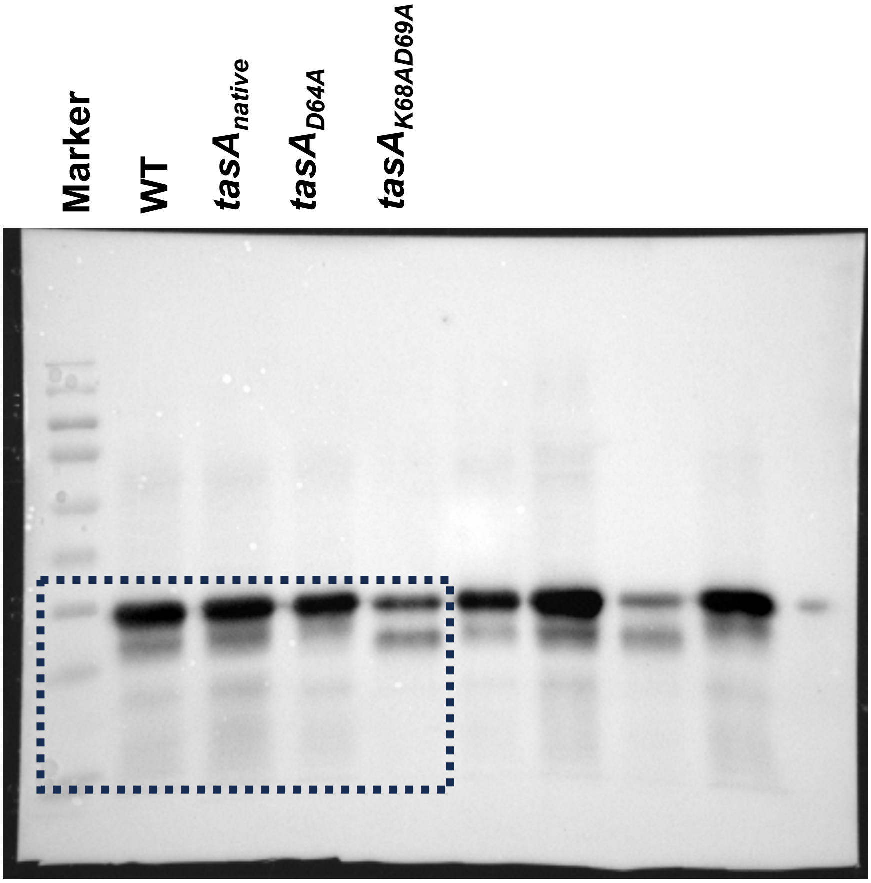

Supplementary figure 4B top left

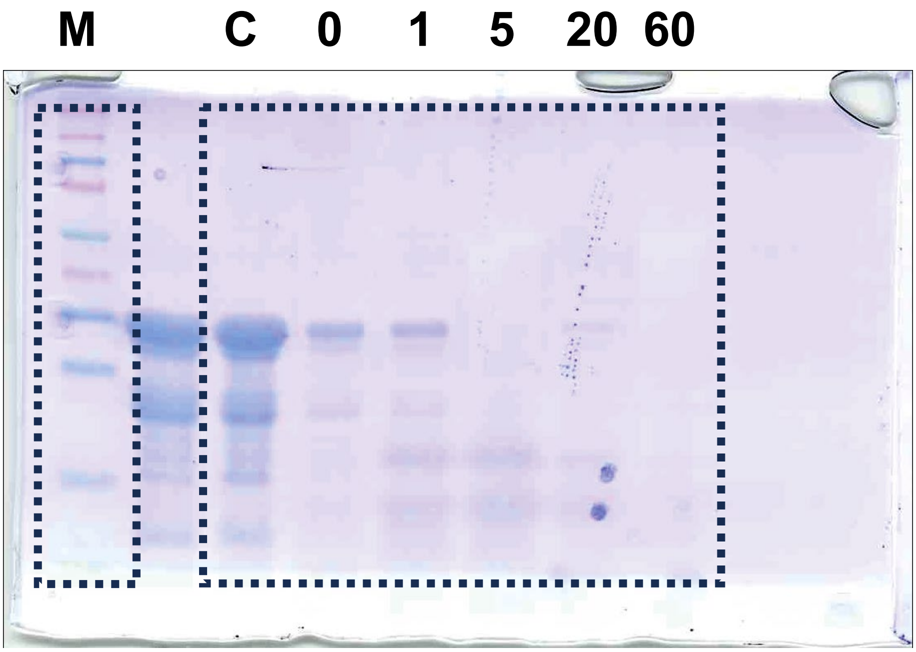

Lanes were loaded with samples corresponding to one digestion time

Supplementary figure 4B top right

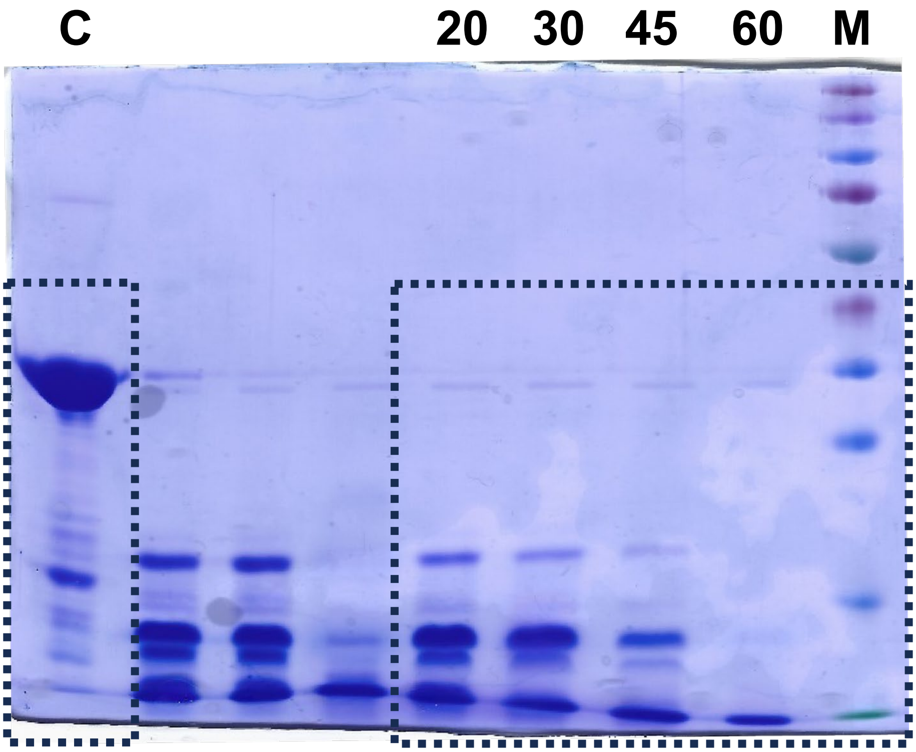

Lanes were loaded with samples corresponding to one digestion time

Supplementary figure 4B bottom left

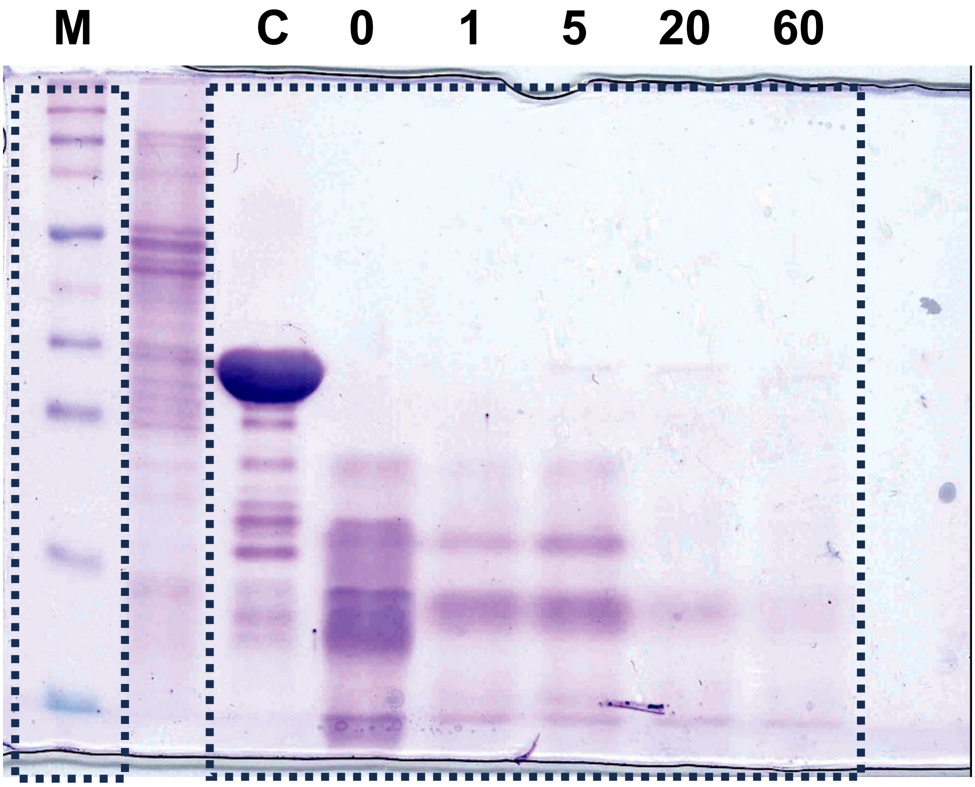

Lanes were loaded with samples corresponding to one digestion time

Supplementary figure 4B bottom right

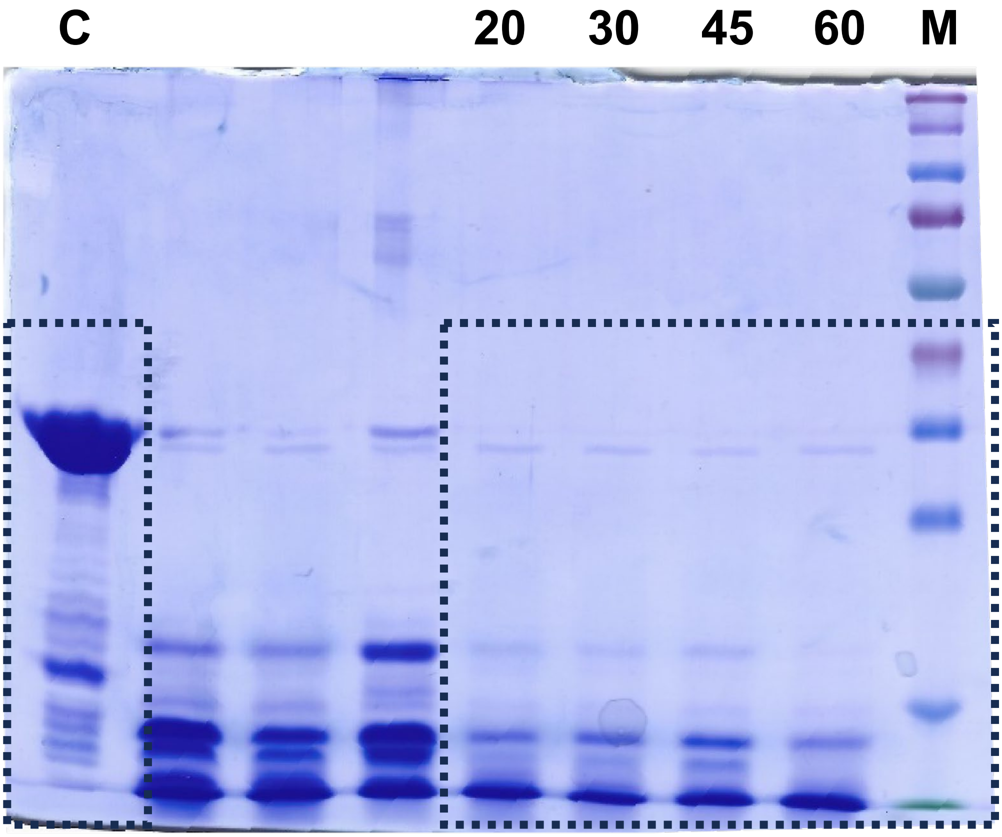

Lanes were loaded with samples corresponding to one digestion time
